# Outcomes after coronary angiography for unstable angina compared to stable angina, myocardial infarction and an asymptomatic general population

**DOI:** 10.1016/j.ijcha.2022.101099

**Published:** 2022-07-31

**Authors:** Kristina Fladseth, Tom Wilsgaard, Haakon Lindekleiv, Andreas Kristensen, Jan Mannsverk, Maja-Lisa Løchen, Inger Njølstad, Ellisiv B Mathiesen, Thor Trovik, Svein Rotevatn, Signe Forsdahl, Henrik Schirmer

**Affiliations:** a Department of Clinical Medicine, UiT The Arctic University of Norway, Tromsø, Norway; b Department of Cardiology, University Hospital of North Norway, Tromsø, Norway; c Department of Community Medicine, UiT The Arctic University of Norway, Tromsø, Norway; d Department of Neurology, University Hospital of North Norway, Tromsø, Norway; e Department of Heart Disease, Haukeland University Hospital, Bergen, Norway; f Norwegian Registry of Invasive Cardiology, Bergen, Norway; g Department of Radiology, University Hospital North Norway, Tromsø, Norway; h Institute of Clinical Medicine, University of Oslo, Lørenskog, Norway; i Department of Cardiology, Akershus University Hospital, Lørenskog, Norway

**Keywords:** High-sensitivity troponins, Non-ST elevation acute coronary syndrome, Non-obstructive coronary artery disease, Prognosis, CABG, Coronary artery bypass graft, CAD, Coronary artery disease, CCTA, Coronary computed tomography angiography, ESC, European Society of Cardiology, FFR, Fractional flow reserve, Hs-cTn, High-sensitivity troponin, ICA, Invasive coronary angiography, MACE, Major cardiovascular events, MI, Myocardial infarction, NORIC, Norwegian Registry of Invasive Cardiology, NSTE-ACS, Non-ST-segment elevation acute coronary syndrome, NSTEMI, Non-ST-segment elevation myocardial infarction, PCI, Percutaneous coronary intervention, SA, Stable angina, STEMI, ST-segment elevation myocardial infarction, UA, Unstable angina

## Abstract

**Background:**

The outcomes of real-world unstable angina (UA) in the high-sensitivity troponin era are unclear. We aimed to investigate the outcomes of UA referred to coronary angiography compared to stable angina (SA), non-ST-segment elevation myocardial infarction (NSTEMI), STEMI and a general population.

**Methods:**

We included the 9,694 patients with no prior coronary artery disease (CAD) referred to invasive or CT coronary angiography from 2013 to 2018 in Northern Norway (51% SA, 12% UA, 23% NSTEMI and 14% STEMI), and 11,959 asymptomatic individuals recruited from the Tromsø Study. We used Cox models to estimate the hazard ratios (HR) for all-cause mortality and major adverse cardiovascular events (MACE), defined as cardiovascular death, MI or obstructive CAD.

**Results:**

The median follow-up time was 2.8 years. The incidence rate of death was 8.5 per 1000 person-years (95 % confidence interval [CI] 8.0–9.0) in the general population, 9.7 (95 % CI 8.3–11.5) in SA, 14.9 (95 % CI 11.4–19.6) in UA, 29.7 (95 % CI 25.6–34.3) in NSTEMI and 36.5 (95 % CI 30.9–43.2) in STEMI. In multivariable adjusted analyses, compared with UA, SA had a 38 % lower risk of death and a non-significant lower risk of MACE (HR 0.62, 95 % CI 0.44–0.89; HR 0.86, 95 % CI 0.66–1.11). NSTEMI had a 2.4-fold higher risk of death (HR 2.39, 95 % CI 1.38–4.14) and a 1.6-fold higher risk of MACE (HR 1.62, 95 % CI 1.11–2.38) compared tox UA during the first year after coronary angiography, but a similar risk thereafter. There was no difference in the risk of death for UA with non-obstructive CAD and obstructive CAD (HR 0.78, 95 % CI 0.39–1.57).

**Conclusion:**

UA had a higher risk of death but a similar risk of MACE compared to SA and a lower 1-year risk of death and MACE compared to NSTEMI.

## 1 Introduction

The diagnosis and management of unstable angina (UA) have changed over the last decade, with increasingly sensitive troponins and coronary CT angiography (CCTA) as an alternative to invasive coronary angiography (ICA) [1], [2], [3]. The implementation of high-sensitivity troponins (hs-cTn) led to a 20 % relative increase in the detection of non-ST-segment elevation myocardial infarction (NSTEMI) and a reciprocal decrease in the diagnosis of UA [4], [5]. This is believed to have significantly improved the outcomes of UA. However, existing studies on the outcomes of UA either report results for an unselected chest pain population in the emergency department, combined results for UA and NSTEMI, apply older, less sensitive troponins and biomarkers to differentiate between UA and NSTEMI, or only include individuals with high-risk features and percutaneous coronary intervention (PCI) [3], [4], [6], [7], [8], [9], [10], [11], [12], [13], [14], [15], [16], [17], [18]. As non-obstructive CAD is highly prevalent and associated with a poorer prognosis than previously believed in both stable angina (SA) and myocardial infarction (MI) [19], [20], [21], [22], [23], [24], the outcomes of all patients with suspected UA, including UA with non-obstructive CAD, are of high interest. Therefore, we aimed to study the outcomes of a real-world population with no prior CAD presenting to CCTA or ICA with clinically suspected UA compared to SA, NSTEMI, ST-segment elevation MI (STEMI) and an asymptomatic general population.

## 2 Methods

### 2.1 Study population

**Fig. 1:**
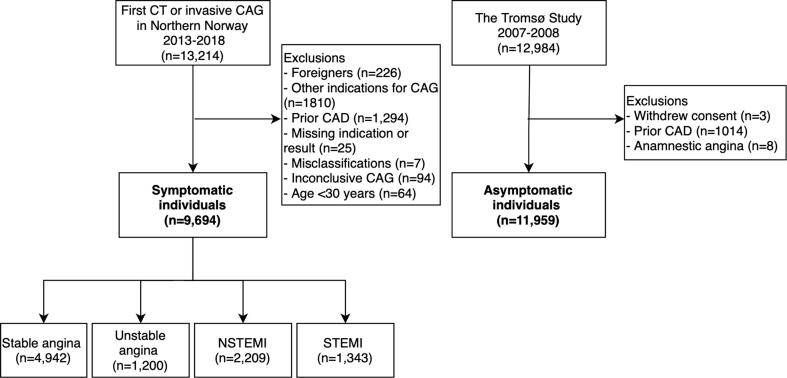
Study population.

This is a registry-based cohort study of patients referred to coronary angiography at the University Hospital of North Norway (UNN) from 1 January 2013 to 31 December 2018. UNN was the sole centre for coronary angiography for the 480,000 inhabitants of Northern Norway. We included patients referred to ICA or CCTA for SA, UA, NSTEMI or STEMI (Fig. 1). Other non-invasive imaging tests for CAD in patients with no prior CAD were generally not applied during the study period in our region.

Patients without a valid personal identification number or not registered as inhabitants in Northern Norway at inclusion were excluded (n = 226). Further, we excluded patients with prior MI or prior revascularization (n = 1294) and patients with other indications for coronary angiography, including pre-operative assessment before heart valve surgery and arrhythmia evaluation (n = 1810), as these patients had a distinctly higher risk of death and MACE. Patients with missing data on the indication, findings or treatment (n = 25) and misclassifications that could not be settled (n = 7) were also excluded. In addition, we excluded 94 patients with inconclusive results on CCTA not followed by an ICA within 180 days. We excluded patients under the age of 30 years (n = 64) to enable comparison with the asymptomatic general population.

As an asymptomatic reference, we recruited individuals from the general population from the sixth survey of the Tromsø Study (Tromsø6) conducted in 2007–2008. The Tromsø Study is a prospective, population-based cohort study in the largest city in Northern Norway [25]. Tromsø6 included 12,984 men and women aged 30 to 87 years old, had 66 % attendance and is described in further detail elsewhere [26]. We excluded 1014 individuals with known CAD based on Tromsø Study data and the coronary angiography registry, including individuals registered with prior MI or prior revascularization at their first coronary angiography. We also excluded eight individuals with self-reported angina on the questionnaire, followed by coronary angiography within 180 days. In addition, three participants withdrew their written consent and were also excluded.

In total, we included 9,694 symptomatic individuals and 11,959 asymptomatic individuals with no prior CAD (Fig. 1). The participants were followed from the date of coronary angiography or the date of enrolment in Tromsø6 until 31 December 2018.

### 2.2 Data collection

The interventional cardiologist or cardiac radiologist recorded data from each consecutive coronary angiography at the time of the procedure. This included prior medical history, risk factors, procedural data, and the indication for coronary angiography. Data from ICA has been recorded from 2005 to 31 April 2013 in a local registry and from 1 May 2013 in the national Norwegian Registry of Invasive Cardiology (NORIC). Data from CCTA has been recorded since the implementation in routine practice in February 2013, first in a local registry, and from 1 January 2016 in NORIC. NORIC has over 99 % coverage for ICA [27]. We found no increase in missing data after transitioning from local registries to a national registry.

In the local registry, admissions with likely misclassifications, such as no obstructive CAD and revascularization, were systematically examined and corrected based on the patient hospital records. NORIC contains predefined constrictions to avoid these misclassifications. Procedures within seven days were included as one admission. To conclude on the overall result of the admission, we systematically reviewed the use of fractional flow reserve (FFR), the extent of CAD, revascularization and the order of the procedures. CCTA with obstructive CAD or inconclusive results followed by ICA within 180 days was replaced by the ICA results. The use of data from both ICA and CCTA reflects the current clinical practice and allows for confirmation of all positive or inconclusive findings on CCTA by ICA.

The Tromsø Study collects data about the study participants through physical examinations, blood samples and self-administered questionnaires. An endpoint committee has verified all incident MIs. Vital status, date of death and cause of death was collected from the National Population Registry and the National Cause of Death Registry, which contains data for all deaths occurring in Norway and abroad for Norwegian citizens. The cause of death is registered using International Classification of Disease 10th revision. Cardiovascular death was defined as codes I00-I99, mainly I20-I25 and I61, I63 and I64. The national personal identification number allowed for linkage on an individual level.

### 2.3 Exposures and covariates

#### 2.3.1 Indication for coronary angiography

The definition of SA, UA, NSTEMI and STEMI was based on the indication for coronary angiography decided by the interventional cardiologist or cardiac radiologist according to ESC guidelines and the universal MI definition [3], [28], [29], [30]. Hs-cTn was implemented in 2009, and the Third Universal MI definition using a rise and/or fall with at least one value over the 99th percentile of hs-cTn to diagnose MI was implemented from 2012 to 2013 [31], [32]. We included only patients from 2013 onwards to comply with the current MI definition [30]. UA was generally defined as acute chest pain at rest, new-onset angina or rapidly worsening angina, and no significant rise/and fall in hs-cTn. Routine sampling of troponin was performed at 0 h and 3 h. UNN and the majority of the other local hospitals use the hs-cTn T assay from Elecsys/Roche (99th percentile upper reference limit of 14 ng/L, coefficient of variation < 10 % at 14 ng/L, level of detection of 5 ng/L).

NORIC contained information on troponin before and after ICA in 70 % of UA patients and 43 % of NSTEMI patients. The local registry had the maximum troponin value before ICA in over 90 % of UA and NSTEMI patients with UNN as their local hospital (34 % of the local registry). The median hs-cTn T value was < 10 ng/L in UA patients (interquartile range [IQR] < 10–18 ng/L), and 209 ng/L in NSTEMI patients (IQR 59–794 ng/L). We did not have data on hs-cTn values under 10 ng/L. In NORIC, we redefined nine UA as NSTEMI based on significantly rising hs-cTn and no PCI.

#### 2.3.2 The extent of coronary artery disease

The extent of CAD was registered per segment by the interventional cardiologist or the cardiac radiologist. Obstructive CAD was defined as ≥ 50 % diameter stenosis of an epicardial coronary artery on ICA [33]. Non-obstructive CAD was defined as 0–49 % diameter stenosis. For invasive coronary angiography, FFR was generally measured with visual diameter stenosis around 40–70 %, and obstructive CAD was defined as FFR below 0.80. The extent of obstructive CAD was further divided into one-vessel (1VD), two-vessel (2VD) and three-vessel (3VD) and/or left main stem disease (LMS).

#### 2.3.3 Covariates

The coronary angiography registries contain information regarding age, sex, smoking status, diabetes, use of lipid-lowering and antihypertensive drugs, body mass index, and kidney function. Overall, there were low rates of missing data for cardiovascular risk factors (0–6 %). Kidney function was reported as estimated glomerular filtration rate (eGFR) and was missing in 10 % of the coronary angiography registry registrations. The local CCTA registry from 2013 to 2015 did not record data on diabetes or drugs.

### 2.4 Outcomes

The primary endpoint was all-cause mortality, and the secondary endpoint was major adverse cardiovascular events (MACE). For the definition of MACE, the following endpoints were available: cardiovascular death, non-fatal MI referred to ICA or new obstructive CAD confirmed by ICA.

### 2.5 Statistical analysis

Baseline characteristics are reported as counts, percentages or means ± standard deviation. Individuals were followed from the date of ICA/CCTA or date of enrolment in the Tromsø Study until the date of death or until the end of the study period, 31 December 2018. The cause of death was only available through 2017. Cumulative incidence was expressed as the number of events per 100 individuals at one and five years. Crude incidence rates (IR) were expressed as the number of events per 1000 person-years at risk. We used Cox proportional hazard regression models to estimate the survival functions and hazard ratios (HR) for all-cause mortality and MACE by indication of ICA/CCTA and extent of CAD. The reference group was UA. Individuals in the general population referred to ICA or CCTA during the study period contributed with person-time as the asymptomatic general population until the date of the ICA/CCTA, after that as symptomatic.

Survival functions for all-cause mortality and MACE were presented adjusted for age and stratified by sex. The HR for all-cause mortality and MACE were estimated in two models; model 1 adjusted for age and sex, and model 2 adjusted for age, sex, smoking, antihypertensive drugs, lipid-lowering drugs, diabetes, BMI and kidney function. Statistical interactions between the exposure variables and sex were tested by including cross-product terms in the models and was significant for the general population and SA. The models are presented stratified by sex in the Supplementary Tables 1-3. The proportional hazard assumption was tested by Schoenfeld residuals. As expected, the assumption was violated as the relative risk of outcomes changed over time. Therefore, the main analysis was also presented in two time periods, from 0 to 1 year and after the first year.

To handle missing data on cardiovascular risk factors, we first assessed if the patient had procedures close in time with available data and imputed this data. Then, the remaining missing data was replaced using multiple imputation. The patients with CCTA had fewer cardiovascular risk factors than patients with ICA, and the multiple imputation was performed separately for these groups.

We applied a two-sided significance level of 5 %. The analysis was performed in Stata 16.1 (StataCorp, Texas, USA).

### 2.6 Ethics

The regional ethics committee and the local data protection official at UNN approved the study. We performed a Data Protection Impact Assessment in accordance with the European Union General Data Protection Regulation. The Tromsø Study is approved by the Norwegian Data Protection Agency and the study participants in Tromsø6 gave informed written consent.

## 3 Results

We included 9,694 symptomatic individuals with no prior CAD that underwent ICA or CCTA for SA (51 %), UA (12 %), NSTEMI (23 %) or STEMI (14 %), and 11,959 asymptomatic individuals with no prior CAD from the general population. UA constituted 25 % of the ACS patients, and this proportion remained stable during the study period (p for trend = 0.40). Baseline characteristics are found in Table 1. The UA patients had a mean age of 61 years, and 61 % were men. The mean age was slightly lower than for SA, NSTEMI and STEMI patients, and the proportion of men was higher for UA patients than SA patients but lower than for NSTEMI and STEMI patients. UA patients had an intermediate level of cardiovascular risk factors compared to SA, NSTEMI and STEMI, and higher than the general population. The proportion of non-obstructive CAD was 65 % for SA, 60 % for UA, 18 % for NSTEMI and 7 % for STEMI patients (Table 1).

**Table 1:** Baseline characteristics of patients referred to coronary angiography in Northern Norway from 2013 to 2018 and a general population from the Tromsø Study.

	**SA** **(n = 4942)**	**UA** **(n = 1200)**	**NSTEMI** **(n = 2209)**	**STEMI** **(n = 1343)**	**Gen. pop.** **(n = 11959)**
Age (yrs)	62 ± 11	61 ± 12	65 ± 12	63 ± 12	57 ± 12
Male gender	53 % (2641)	61 % (7 3 3)	67 % (1475)	74 % (9 9 0)	45 % (5372)
Current smoker	18 % (8 5 8)	25 % (2 8 0)	31 % (6 6 1)	43 % (5 1 4)	27 % (3205)
Former smoker	47 % (2227)	40 % (4 5 0)	39 % (8 1 8)	29 % (3 4 4)	36 % (4241)
Use of antihypertensive drugs	49 % (2144)	41 % (4 7 4)	45 % (9 8 4)	31 % (4 1 2)	20 % (2318)
Use of lipid-lowering drugs	58 % (2567)	41 % (4 7 4)	36 % (7 8 6)	15 % (2 0 4)	9 % (1070)
Diabetes mellitus	13 % (5 9 1)	12 % (1 3 5)	14 % (3 2 0)	9 % (1 2 1)	8 % (8 9 3)
BMI (kg/m^2^)	27 ± 5	28 ± 5	27 ± 5	27 ± 4	26 ± 4
Estimated GFR (mL/min/1.73 m^2^)	82 ± 18	85 ± 18	82 ± 20	85 ± 19	93 ± 15
Angiographic characteristics at admission					
Coronary angiography					
ICA	59 % (2926)	93 % (1114)	100 % (2209)	100 % (1343)	
CCTA + ICA	16 % (7 7 2)	1 % (13)			
CCTA	25 % (1244)	6 % (73)			
Extent of CAD					
Non-obCAD^a^	65 % (3232)	60 % (7 1 7)	18 % (4 0 3)	7 % (90)	
1VD	18 % (8 7 2)	21 % (2 5 3)	42 % (9 3 3)	56 % (7 5 2)	
2VD	8 % (4 1 4)	10 % (1 1 5)	21 % (4 6 6)	23 % (3 1 0)	
3VD/LMS	9 % (4 2 4)	10 % (1 1 5)	18 % (4 0 7)	14 % (1 9 1)	
FFR	7 % (3 4 5)	7 % (83)	6 % (1 3 0)	2 % (23)	
Revascularization^b^	27 % (1337)	38 % (4 5 4)	78 % (1717)	92 % (1230)	
PCI	20 % (1010)	30 % (3 6 6)	69 % (1514)	89 % (1191)	
CABG	7 % (3 6 6)	8 % (97)	11 % (2 5 2)	5 % (61)	

Values are % (*n*) or mean ± SD. BMI indicates body mass index; CABG, coronary artery bypass graft; CAD, coronary artery disease; CCTA, coronary computed tomography angiography; FFR, fractional flow reserve; GFR, glomerular filtration rate; non-obCAD, non-obstructive CAD; NSTEMI, non-ST segment elevation myocardial infarction; PCI, percutaneous coronary intervention; SA, stable angina; STEMI, ST-segment elevation myocardial infarction; UA, unstable angina; 1VD, one-vessel disease; 2VD, two-vessel disease; 3VD/LMS, three-vessel disease and/or left main stem disease.

a Including the participants deferred after coronary CT angiography.

b There is a small overlap in patients receiving both PCI and CABG for revascularization.

### 3.1 All-cause mortality

**Fig. 2:**
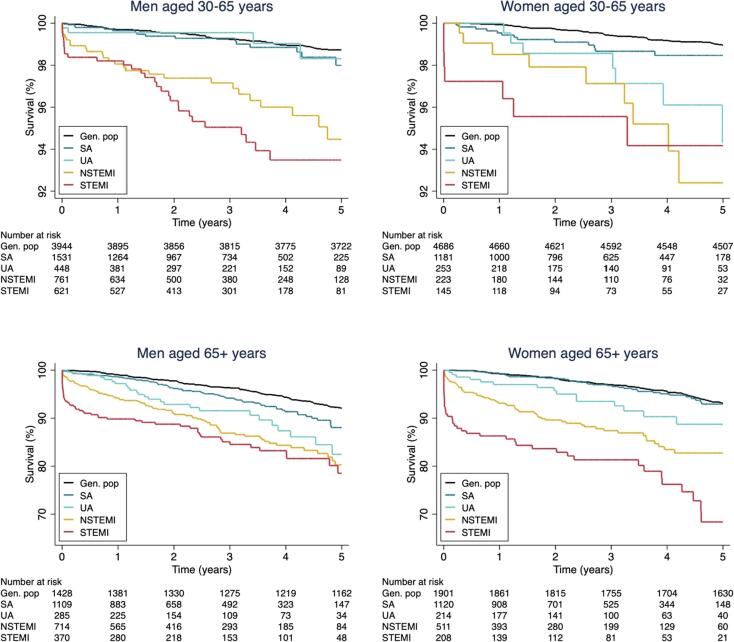
Survival function for all-cause mortality in patients referred to coronary angiography compared to a general population.

There were 511 (5.3 %) deaths during a median follow-up time of 2.8 years (IQR 1.3–4.4) for patients referred to coronary angiography. Cardiovascular disease was the cause of death in 33 % of the patients. Survival functions for all-cause death for SA, UA, NSTEMI, STEMI and the general asymptomatic population are shown in Fig. 2 and Supplementary Fig. 1. The mortality of UA and SA patients was similar to the general population during the first year, while NSTEMI and STEMI patients had higher mortality. After the first year, SA and UA had higher mortality than the general population, and there was less difference in mortality between the different presentations of CAD with more parallel curves.

IR and HR of all-cause mortality for UA compared to SA, NSTEMI, STEMI and the asymptomatic general population are shown in Tables 2 and 3. The IR for death was 8.5 per 1000 person-years (95 % confidence interval [CI] 8.0–9.0) in the general population, 9.7 (95 % CI 8.3–11.5) in SA patients, 14.9 (95 % CI 11.4–19.6) in UA patients, 29.7 (95 % CI 25.6–34.3) in NSTEMI patients and 36.5 (95 % CI 30.9–43.2) in STEMI patients. Cumulative 1-year and 5-year mortality rates are presented in Supplementary Table 4.

**Table 2:** Incidence rates (IR) and hazard ratios (HR) with 95% confidence intervals (CI) for all-cause mortality for patients referred to coronary angiography and a general population.

**All-cause mortality**	**Events**	**Person-years**	**Crude IR** **(95 % CI)**^a^	**Age- and sex-adjusted** **HR (95 % CI)**	**Multivariable adjusted** **HR (95 % CI)**^b^
General population	980	115,463	8.5 (8.0–9.0)	0.55 (0.41–0.74)	0.54 (0.39–0.76)
SA	140	14,379	9.7 (8.3–11.5)	0.66 (0.48–0.90)	0.62 (0.44–0.89)
UA	53	3548	14.9 (11.4–19.6)	Ref.	Ref.
NSTEMI	182	6138	29.7 (25.6–34.3)	1.34 (0.98–1.82)	1.26 (0.90–1.78)
STEMI	136	3726	36.5 (30.9–43.2)	2.11 (1.53–2.89)	1.62 (1.10–2.37)

SA indicates stable angina; UA, unstable angina; NSTEMI, non-ST elevation myocardial infarction; STEMI, ST-elevation myocardial infarction.

a Per 1000 person-years.

b Adjusted for age, sex, smoking status, antihypertensive drugs, lipid-lowering drugs, diabetes, BMI and kidney function.

**Table 3:** Incidence rates (IR) and hazard ratios (HR) with 95 % confidence intervals (CI) for all-cause mortality for patients referred to coronary angiography and a general population at 0–1 year and after 1 year.

	**0**–**1 year**			**>1 year**		
**All-cause mortality**	**Crude** **IR (95 % CI)**^a^	**Age- and sex-adjusted** **HR (95 % CI)**	**Multivariable adjusted** **HR (95 % CI)**^b^	**Crude** **IR (95 % CI)**^a^	**Age- and sex-adjusted** **HR (95 % CI)**	**Multivariable adjusted** **HR (95 % CI)**^b^
General population	3.6 (2.7–4.9)	0.37 (0.21–0.68)	0.48 (0.24–0.95)	9.0 (8.5–9.6)	0.56 (0.40–0.78)	0.52 (0.36–0.76)
SA	6.9 (4.8–9.8)	0.50 (0.27–0.93)	0.53 (0.26–1.06)	11.0 (9.2–13.3)	0.71 (0.49–1.03)	0.65 (0.43–0.97)
UA	13.6 (8.2–22.6)	Ref.	Ref.	15.5 (11.3–21.4)	Ref.	Ref.
NSTEMI	45.1 (36.6–55.5)	2.39 (1.38–4.14)	2.47 (1.30–4.71)	22.3 (18.2–27.4)	0.97 (0.66–1.41)	0.90 (0.60–1.36)
STEMI	68.0 (54.6–84.7)	4.44 (2.56–7.71)	3.84 (1.95–7.57)	22.0 (16.9–28.5)	1.22 (0.81–1.85)	0.97 (0.60–1.57)

SA indicates stable angina; UA, unstable angina; NSTEMI, non-ST elevation myocardial infarction; STEMI, ST-elevation myocardial infarction.

a Per 1000 person-years.

b Adjusted for age, sex, smoking status, antihypertensive drugs, lipid-lowering drugs, diabetes, BMI and kidney function.

In multivariable adjusted analyses, the risk of death compared to UA patients was 46 % lower in the general population (HR 0.54, 95 % CI 0.39–0.76), 38 % lower in SA patients (HR 0.62, 95 % CI 0.44–0.89), non-significant higher in NSTEMI (HR 1.26, 95 % CI 0.90–1.78) and 62 % higher in STEMI patients (HR 1.62, 95 % CI 1.10–2.37) (Table 2). These findings were similar in analyses stratified by sex (Supplementary Table 2). The 1-year risk after ICA/CCTA compared to UA patients was lower in the general population (HR 0.48, 95 % CI 0.24–0.95), non-significant lower in SA patients (HR 0.53, 95 % CI 0.26–1.06), 2.5-fold higher in NSTEMI patients (HR 2.47, 95 % CI 1.30–4.71), and 4-fold higher in STEMI patients (HR 3.84, 95 % CI 1.95–7.57) (Table 3).

### 3.2 Major adverse cardiovascular events

The secondary endpoint of MACE occurred in 811 (8.4 %) patients referred to coronary angiography, of which cardiovascular death constituted 19 % (n = 152), MI 26 % (n = 211), and obstructive CAD 55 % (n = 448). The IR and HR for MACE for UA compared to SA, NSTEMI, STEMI and the asymptomatic general population are shown in Tables 4 and 5. Survival functions and cumulative 1-year and 5-year incidence of MACE are presented in Supplementary Fig. 2 and Supplementary Table 2. The IR of MACE per 1000 person-years was 8.1 (95 % CI 7.6–8.6) in the asymptomatic general population, 21.8 (95 % CI 19.5–24.4) in SA patients, 23.5 (95 % CI 18.9–29.2) in UA patients, 44.0 (9 %% CI 38.9–49.8) in NSTEMI patients and 51.6 (95 % CI 44.6–59.7) in STEMI patients. In multivariable adjusted analyses, the risk of MACE compared to UA patients was 50 % lower in the general population (HR 0.50, 95 % CI 0.39–0.64), similar in SA patients (HR 0.86, 95 % CI 0.66–1.11), 38 % higher in NSTEMI (HR 1.38, 95 % CI 1.06–1.80) and 91 % higher in STEMI patients (HR 1.91, 95 % CI 1.42–2.57) (Table 4). These findings were similar in analyses stratified by sex (Supplementary Table 3). During the first year after coronary angiography, the risk of MACE compared to UA patients was still lower in the general population (HR 0.32, 95 % CI 0.21–0.48), non-significant lower in SA patients (HR 0.77, 95 % CI 0.53–1.13), 62 % higher in NSTEMI patients (HR 1.62, 95 % CI 1.11–2.38), and 3-fold higher in STEMI (HR 2.85, 95 % CI 1.91–4.12) (Table 5).

**Table 4:** Incidence rates (IR) and hazard ratios (HR) with 95% confidence intervals (CI) for major adverse cardiovascular events (MACE) for patients referred to coronary angiography and a general population.

**MACE**	**Events**	**Person-years**	**Crude IR** **(95 % CI)**^a^	**Age- and sex-adjusted HR (95 % CI)**	**Multivariable adjusted** **HR (95 % CI)**^b^
General population	936	115,384	8.1 (7.6–8.6)	0.49 (0.39–0.63)	0.50 (0.39–0.64)
SA	301	13,801	21.8 (19.5–24.4)	0.93 (0.73–1.19)	0.86 (0.66–1.11)
UA	80	3406	23.5 (18.9–29.2)	Ref	Ref.
NSTEMI	251	5701	44.0 (38.9–49.8)	1.44 (1.12–1.85)	1.38 (1.06–1.80)
STEMI	179	3470	51.7 (44.6–59.8)	1.88 (1.44–2.45)	1.91 (1.42–2.57)

SA indicates stable angina; UA, unstable angina; NSTEMI, non-ST elevation myocardial infarction; STEMI, ST-elevation myocardial infarction. MACE is defined as cardiovascular death or MI or new obstructive CAD on coronary angiography.

a Per 1000 person-years.

b Adjusted for age, sex, smoking status, antihypertensive drugs, lipid-lowering drugs, diabetes, BMI and kidney function.

**Table 5:** Incidence rates (IR) and hazard ratios (HR) with 95 % confidence intervals (CI) for major adverse cardiovascular events (MACE) for patients referred to coronary angiography and a general population at 0–1 year and after 1 year.

	**0**–**1 year**			**>1 year**		
**MACE**	**Crude** **IR (95 % CI)**^a^	**Age- and sex-adjusted** **HR (95 % CI)**	**Multivariable adjusted** **HR (95 % CI)**^b^	**Crude** **IR (95 % CI)**^a^	**Age- and sex-adjusted** **HR (95 % CI)**	**Multivariable adjusted** **HR (95 % CI)**^b^
General population	8.2 (6.8–10.0)	0.29 (0.20–0.43)	0.32 (0.21–0.48)	8.1 (7.6–8.7)	0.61 (0.44–0.84)	0.59 (0.42–0.82)
SA	28.1 (23.6–33.5)	0.79 (0.55–1.13)	0.77 (0.53–1.13)	18.8 (16.2–21.8)	1.05 (0.75–1.48)	0.92 (0.65–1.30)
UA	35.9 (26.2–49.1)	Ref.	Ref.	17.7 (13.0–24.0)	Ref.	Ref.
NSTEMI	70.3 (59.4–83.2)	1.63 (1.14–2.34)	1.62 (1.11–2.38)	30.7 (25.6–36.8)	1.29 (0.90–1.85)	1.22 (0.85–1.77)
STEMI	107 (89.6–128)	2.68 (18.7–3.85)	2.85 (1.91–4.27)	24.5 (18.9–31.7)	1.15 (0.77–1.73)	1.16 (0.74–1.80)

SA indicates stable angina; UA, unstable angina; NSTEMI, non-ST elevation myocardial infarction; STEMI, ST-elevation myocardial infarction. MACE is defined as cardiovascular death or MI or new obstructive CAD on coronary angiography.

a Per 1000 person-years.

b Adjusted for age, sex, smoking status, antihypertensive drugs, lipid-lowering drugs, diabetes, BMI and kidney function.

### 3.3 The extent of coronary artery disease

**Fig. 3:**
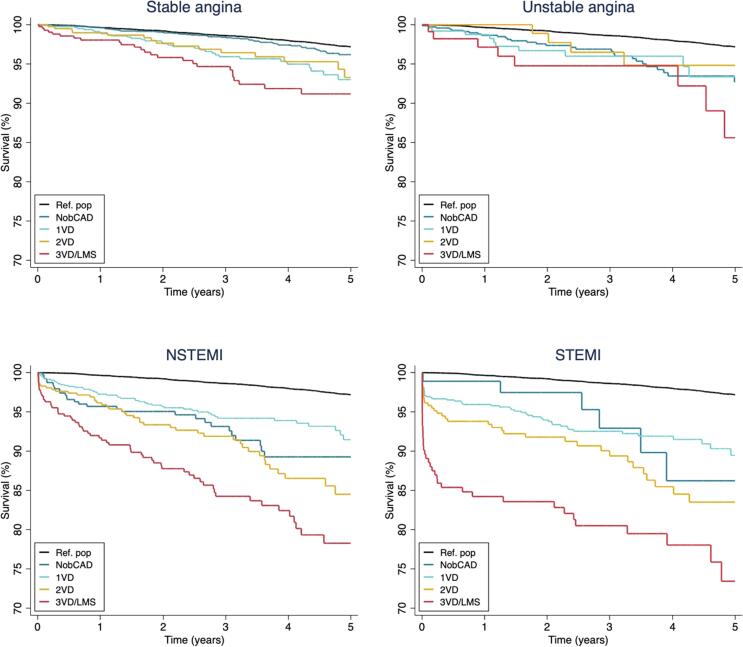
Survival functions for all-cause mortality for patients referred to coronary angiography by the extent of coronary artery disease. CAD indicates coronary artery disease; non-obCAD, non-obstructive CAD; NSTEMI, non-ST segment elevation myocardial infarction; STEMI, ST-segment elevation myocardial infarction; 1VD, one-vessel disease; 2VD, two-vessel disease; 3VD/LMS, three-vessel disease and/or left main stem disease.

Survival for all-cause death by indication and extent of CAD is shown in Fig. 3. The mortality rate in UA patients with non-obstructive CAD and obstructive CAD was 14.1 (95 % CI 9.9–20.2) and 16.2 (95 % CI 10.8–24.4) per 1000 person-years, respectively. In multivariable adjusted analyses, there was no difference in risk of death among UA patients with non-obstructive CAD and obstructive CAD (HR 0.78, 95 % CI 0.39–1.57). Among patients with obstructive CAD, the risk of death compared to UA patients was not significantly different in SA (HR 0.78, 95 % CI 0.47–1.29), non-significantly higher in NSTEMI (HR 1.50, 95 % CI 0.93–2.41), and higher in STEMI (HR 1.90, 95 % CI 1.15–3.14).

The IR of MACE in UA patients with non-obstructive CAD and obstructive CAD was 8.6 (95 % CI 5.4–13.6) and 46.1 (95 % CI 35.8–59.4) per 1000 person-years, respectively. In multivariable adjusted analyses, UA patients with obstructive CAD had a 5-fold higher risk of MACE than UA patients with non-obstructive CAD (HR 4.73, 95 % CI 2.45–9.16). This was mainly driven by higher rates of new obstructive CAD in UA patients with obstructive CAD. Among patients with obstructive CAD, there was no difference in risk of MACE between the different clinical presentations, SA, UA, NSTEMI and STEMI (Supplementary Fig. 3).

## 4 Discussion

In our real-world registry-based study of patients with no prior CAD, we found that UA patients had a higher risk of death but no significant difference in the risk of MACE compared to SA patients, and half the risk of death and MACE compared to NSTEMI patients during the first year after coronary angiography. This aligns with the increasing evidence that UA in the hs-cTn era is associated with a better prognosis than NSTEMI and a more similar prognosis to SA [3], [4], [11], [12], [13], [14], [15], [16], [17], [18]. However, the other studies in the hs-cTn era either only include patients that underwent PCI [11], patients with high-risk criteria [13], small populations [14], or unselected chest pain populations presenting to emergency departments [3], [4], [12], [15], [16], [17], [18]. Most studies also only report up to 1-year outcome data [13], [14]. Further, we have focused only on patients with no prior CAD, as patients with established CAD had a distinctly poorer prognosis. Therefore, our study adds to the existing knowledge.

We report lower outcomes rates for UA patients compared to other studies [3], [4], [11], [13], [14]. In addition to no prior CAD, our UA population had a high proportion of non-obstructive CAD and few patients with chronically elevated hs-cTn, making it a relatively low-risk population. Further, we have an overall low rate of outcomes in our population, especially in SA and UA patients, compared to a general reference population. The High-STEACS, APACE and RAPID-CPU studies reported a 1-year incidence of death and MI in UA patients of 3–4 % and 3–11 % respectively [13], [14], compared to our 1-year incidences of 1.4 % for death and MACE. These studies had a high rate of prior CAD and other risk factors likely contributing to this [13], [14]. Further, these studies retrospectively defined UA from larger chest pain populations. Comparable to our study, 34 % of the 280 patients defined as having UA in the RAPID-CPU underwent PCI or CABG [14]. However, in High-STEACS and APACE, only half of the UA patients were referred to coronary angiography, yet 95 % of the referred patients had obstructive CAD, a higher proportion than that of NSTEMI patients in these populations. This discrepancy between how many referred to coronary angiography and the very high rate of obstructive CAD makes the results of their overall UA population difficult to interpret [13].

The definition of UA is challenging in research and clinical practice as there is no universal definition. UA is a substantial part of ACS in our study, being the indication for 25 % of acute coronary angiographies and 13 % of acute revascularizations, which is comparable to, or higher, than previous studies [3], [10], [13], [14], [34]. Selecting UA patients from the chest pain/suspected ACS population in emergency departments is based on symptoms suspected to be caused by myocardial ischemia and no myocardial injury evident by a significant rise or fall in troponin. The outcomes of the unselected chest pain population are well researched in several studies on the ESC 0 h/ 1 h and 0 h/2h hs-cTn algorithms for rule-out, observe, and rule-in for MI, with a 1-year cumulative incidence of death of 0.0–2.2 %, 4.0–7.6 % and 9.8–16.1 %, respectively [3], [12], [15], [16], [17], [18]. UA patients may be present in all groups, yet our results are comparable to the rule-out group, indicating a very favourable short-term prognosis for our UA population with no prior CAD. This was despite our UA population being defined by the interventional cardiologists' clinical suspicion of UA and decision to perform coronary angiography. Our findings support the 2020 ESC Acute Coronary Syndrome without Persistent ST-segment Elevations Guidelines, focusing on detecting the individuals with NSTEMI that have a significantly worse prognosis and a more individual workup for patients with suspected UA [3]. The guidelines further recommend using more CCTA to exclude CAD in patients presenting with acute chest pain. The high prevalence of non-obstructive CAD and favourable prognosis for UA in our population support this.

However, despite the overall favourable prognosis, UA patients, and especially UA patients with non-obstructive CAD, cannot be dismissed as a benign condition. We found a similar risk of death among UA patients with obstructive CAD and non-obstructive CAD. Further, UA patients had a higher risk of death than SA patients and a similar risk of death as NSTEMI after the first year. This may support performing CCTA on a relatively low threshold. In the current guidelines on the management of UA, how to select patients for CCTA or other non-invasive imaging remains unclear. Further research on this topic is warranted.

Finally, we also observed that NSTEMI patients did not have a higher long-term risk of death and MACE compared to STEMI patients as most other cohort studies report [34], [35], [36], [37]. However, a study based on the PCI registry in Sweden also found no difference in the risk of death and MI between NSTEMI and STEMI [10]. A possible explanation is that our study only included patients with no prior CAD and the study from Sweden adjusted for prior CAD [10]. Further, the implementation of increasingly sensitive troponins diagnose NSTEMI in patients that previously would be diagnosed with UA. These patients have a better prognosis than the other NSTEMI patients and may affect the overall outcomes of NSTEMI compared to STEMI [5]. However, this was outside the scope of this article and further investigation is needed.

## 5 Limitations

There are some limitations to our study population, including being recruited from a single centre. The classification of SA, UA, NSTEMI and STEMI was based on the interventional cardiologists’ presumed diagnosis before the ICA/CCTA and not the final diagnosis. We chose to reclassify-nine patients from UA to NSTEMI due to significantly rising troponin and no PCI. We did not reclassify UA patients that underwent PCI as this could be related to the procedure. We did not have available troponin values for all patients, and only recordings of the initial troponin value at presentation and the maximum troponin value of the admission. Improved data on troponin would be useful, and allow us to better ensure that UA and NSTEMI were classified correctly. However, this affected a minority of our study population, and we do not think it has affected our results.

We had a high rate of non-obstructive CAD for all indications of coronary angiography. However, other studies report up to 62 % non-obstructive CAD for SA and 14 % for MI combined, similar to our results [19], [21], [23]. We believe our study population is representative of other coronary angiography populations with no prior CAD met in clinical practice in high-resource health care systems. We did not have data on the degree of non-obstructive CAD. Further, we did not have sufficient data on left ventricular ejection fraction or medication at discharge.

National registries ensure near-complete follow-up data for the outcomes. However, an individual would be lost to follow-up if the coronary angiography was performed abroad or in another region of Norway before NORIC had full national coverage and lost to follow-up for death if both emigrated from Norway and no longer registered as a Norwegian citizen. We believe this is unlikely to have affected our results. The cause of death was only available through 2017, so the MACE data is not complete for 2018. However, sensitivity analyses for MACE from 2013 to 2017 demonstrated similar risk estimates.

Despite our UA population being larger than the other studies in the hs-cTn era, we cannot exclude the risk of type II errors, especially for the non-significant different risk of death between UA with obstructive and non-obstructive CAD, and non-significant different risk of MACE between UA and SA. We also did not have data on the individuals with CAD that did not undergo coronary angiography, including no data on MI as endpoint if not referred to coronary angiography. However, coronary angiography was performed in around 90 % of STEMI patients and about 70 % of NSTEMI patients under 85 years old, with only slightly lower rates in women reducing the risk of bias [38]. Further, the asymptomatic general population was recruited from 2007 to 2008, while the symptomatic population was recruited from 2013 to 2018. As the incidence of MI and cardiovascular death is declining in Norway this may overestimate the incidence rate of death and MACE in the general population, and underestimate the risk of SA, UA and MI compared to the general population.

## 6 Conclusions

In a real-world population presenting to coronary angiography with no prior CAD, we found that UA patients had a higher risk of death but no significant difference in the risk of MACE compared to SA patients, and a lower 1-year risk of death and MACE than NSTEMI patients, but not after the first year. Unstable angina patients with non-obstructive CAD had a similar risk of death as UA patients with obstructive CAD.

## Declaration of Competing Interest

The authors declare that they have no known competing financial interests or personal relationships that could have appeared to influence the work reported in this paper.
