# Supplementary material for: Outcomes after coronary angiography for unstable angina compared to stable angina, myocardial infarction and an asymptomatic general population
Source: Int J Cardiol Heart Vasc. 2022 Jul 31;42:101099. doi: 10.1016/j.ijcha.2022.101099 (PMC9352908; doi:10.1016/j.ijcha.2022.101099)
Supplement: Supplementary data 1 [file mmc1.docx]

## Supplementary Table 1. Baseline characteristics of men and women referred to coronary angiography in Northern Norway from 2013 to 2018 compared to a general population from the Tromsø Study.

|  |  | |  | |  | |  | |  | |
| --- | --- | --- | --- | --- | --- | --- | --- | --- | --- | --- |
|  |  | |  | |  | |  | |  | |
|  | **SA** | | **UA** | | **NSTEMI** | | **STEMI** | | **Gen. pop.** | |
|  | Men  (n=2641) | Women  (n=2301) | Men  (n=733) | Women  (n=467) | Men  (n=1475) | Women  (n=734) | Men  (n=990) | Women  (n=353) | Men  (n=5372) | Women  (n=6587) |
|  |  | |  | |  | |  | |  | |
|  |  | |  | |  | |  | |  | |
| Age (yr) | 61±11 | 63±11 | 61±12 | 62±12 | 64±12 | 69±12 | 61±11 | 68±13 | 56±12 | 57±13 |
| Current smoker | 18% (464) | 18% (394) | 27% (185) | 22% (95) | 32% (463) | 29% (198) | 41% (362) | 51% (152) | 27% (1433) | 27% (1772) |
| Former smoker | 50% (1261) | 44% (966) | 43% (298) | 35% (152) | 40% (576) | 35% (242) | 32% (284) | 20% (60) | 39% (2060) | 34% (2181) |
| Use of anti-hypertensive drugs | 48% (1177) | 49% (967) | 40% (287) | 42% (187) | 43% (628) | 49% (356) | 28% (278) | 39% (134) | 18% (982) | 21% (1336) |
| Use of lipid-lowering drugs | 61% (1480) | 55% (1087) | 39% (280) | 44% (194) | 34% (500) | 39% (286) | 15% (144) | 11% (37) | 9% (462) | 9% (608) |
| Diabetes mellitus | 14% (347) | 12% (244) | 12% (89) | 10% (46) | 14% (200) | 16% (120) | 9% (84) | 8% (29) | 8% (400) | 8% (493) |
| BMI (kg/m^2^) | 28±4 | 27±5 | 28±5 | 27±5 | 27±5 | 26±5 | 27±4 | 26±5 | 27±4 | 26±5 |
| Estimated GFR (mL/min/1.73m^2^) | 83±18 | 82±18 | 86±17 | 85±18 | 85±19 | 77±20 | 87±18 | 79±21 | 94±14 | 93±15 |
| Angiographic findings |  |  |  |  |  |  |  |  |  |  |
| Non-obCAD^a^ | 54% (1421) | 79% (1811) | 49% (356) | 77% (361) | 12% (177) | 31% (226) | 6% (61) | 8% (29) |  |  |
| 1VD | 22% (581) | 13% (291) | 27% (197) | 12% (56) | 44% (656) | 38% (277) | 57% (562) | 54% (190) |  |  |
| 2VD | 12% (304) | 5% (110) | 12% (90) | 5% (25) | 24% (356) | 15% (110) | 23% (232) | 22% (78) |  |  |
| 3VD/LMS | 13% (335) | 4% (89) | 12% (90) | 5% (25) | 19% (286) | 16% (121) | 14% (135) | 16% (56) |  |  |
| Revascularization^b^ | 37% (970) | 16% (367) | 49% (358) | 21 (96) | 84% (1245) | 64% (472) | 92% (915) | 89% (315) |  |  |
| PCI | 26% (696) | 14% (314) | 39% (286) | 17% (80) | 73% (1080) | 59% (434) | 89% (886) | 86% (305) |  |  |
| CABG | 12% (306) | 3% (60) | 11% (77) | 4% (20) | 13% (191) | 7% (51) | 5% (45) | 5% (16) |  |  |
| FFR etc. | 9% (306) | 5% (118) | 8% (60) | 3% (23) | 6% (94) | 5% (36) | 2% (19) | 1% (4) |  |  |
| CCTA | 37% (965) | 46% (1051) | 5% (40) | 10% (46) |  |  |  |  |  |  |
|  |  | |  | |  | |  | |  | |
|  |  | |  | |  | |  | |  | |

Values are % (*n*) or mean±SD. BMI indicates body mass index; CABG, coronary artery bypass graft; CAD, coronary artery disease; CCTA, coronary computed tomography angiography; FFR, fraction flow reserve; GFR, glomerular filtration rate; non-obCAD, non-obstructive CAD; NSTEMI, non-ST segment elevation myocardial infarction; PCI, percutaneous coronary intervention; STEMI, ST-segment elevation myocardial infarction; 1VD, one-vessel disease; 2VD, two-vessel disease; 3VD/LMS, three-vessel disease and/or left main stem disease.

^a^Including the participants deferred after coronary CT angiography.

^b^There is a small overlap in patients receiving both PCI and CABG for revascularisation.

## Supplementary Table 2. Incidence rates (IR) and hazard ratios (HR) with 95% confidence intervals (CI) for all-cause mortality by sex and indication for coronary angiography

|  |  |  |  |  |  |  |  |  |
| --- | --- | --- | --- | --- | --- | --- | --- | --- |
|  |  |  |  |  |  |  |  |  |
| **All-cause mortality** | **Events** | | **Person-years** | | **Age-adjusted**  **IR (95% CI)^a^** | | **Multivariable adjusted**  **HR (95% CI)^b^** | |
|  |  |  |  |  |  |  |  |  |
|  |  |  |  |  |  |  |  |  |
|  | Men | Women | Men | Women | Men | Women | Men | Women |
|  |  |  |  |  |  |  |  |  |
|  |  |  |  |  |  |  |  |  |
| General population | 4777 | 503 | 50914 | 64549 | 11.4 (10.7-12.1) | 12.9 (12.1-13.8) | 0.60 (0.39-0.94) | 0.46 (0.28-0.75) |
| Stable angina | 88 | 53 | 7537 | 6845 | 8.7 (7.3-10.1) | 9.9 (8.2-11.5) | 0.81 (0.51-1.28) | 0.42 (0.24-0.74) |
| Unstable angina | 31 | 22 | 2110 | 1438 | 13.4 (9.8-17.1) | 15.2 (11.1-19.4) | Ref. | Ref. |
| NSTEMI | 112 | 70 | 4180 | 1958 | 18.4 (15.7-21.1) | 20.9 (17.8-23.9) | 1.38 (0.88-2.15) | 1.12 (0.66-1.92) |
| STEMI | 85 | 51 | 2779 | 941 | 29.3 (24.4-34.2) | 33.2 (27.7-38.8) | 1.55 (0.95-2.55) | 1.80 (0.98-3.31) |
|  |  |  |  |  |  |  |  |  |
|  |  |  |  |  |  |  |  |  |

NSTEMI indicates non-ST elevation myocardial infarction; STEMI, ST-elevation myocardial infarction.

^a^Per 1000 person-years.

^b^Adjusted for age, smoking status, antihypertensive drugs, lipid-lowering drugs, diabetes, BMI and kidney function.

## Supplementary Table 3. Incidence rates (IR) and hazard ratios (HR) with 95% confidence intervals (CI) for major adverse cardiovascular events (MACE) by sex and indication for coronary angiography

|  |  |  |  |  |  |  |  |  |
| --- | --- | --- | --- | --- | --- | --- | --- | --- |
|  |  |  |  |  |  |  |  |  |
| **MACE** | **Events** | | **Person-years** | | **Age-adjusted**  **IR (95% CI)^a^** | | **Multivariable adjusted**  **HR (95% CI)^b^** | |
|  |  |  |  |  |  |  |  |  |
|  |  |  |  |  |  |  |  |  |
|  | Men | Women | Men | Women | Men | Women | Men | Women |
|  |  |  |  |  |  |  |  |  |
|  |  |  |  |  |  |  |  |  |
| General population | 587 | 349 | 50874 | 64510 | 11.4 (10.7-12.2) | 13.0 (12.1-13.8) | 0.50 (0.37-0.67) | 0.50 (0.31-0.81) |
| Stable angina | 212 | 89 | 7122 | 6682 | 9.1 (7.6-10.7) | 10.4 (8.7-12.1) | 0.91 (0.67-1.22) | 0.77 (0.47-1.27) |
| Unstable angina | 59 | 21 | 2009 | 1397 | 14.4 (10.5-18.2) | 16.3 (11.9-20.7) | Ref. | Ref. |
| NSTEMI | 178 | 73 | 3867 | 1835 | 19.6 (16.7-22.5) | 22.2 (19.0-25.5) | 1.33 (0.98-1.80) | 1.53 (0.91-2.57) |
| STEMI | 132 | 47 | 2574 | 890 | 30.8 (25.6-35.9) | 34.9 (29.0-40.8) | 1.73 (1.23-2.43) | 2.59 (1.43-4.69) |
|  |  |  |  |  |  |  |  |  |
|  |  |  |  |  |  |  |  |  |

NSTEMI indicates non-ST elevation myocardial infarction; STEMI, ST-elevation myocardial infarction. MACE is defined as repeat angiography with obstructive CAD and/or MI, or cardiovascular death.

^a^Per 1000 person-years.

^b^Adjusted for age, smoking status, antihypertensive drugs, lipid-lowering drugs, diabetes, BMI and kidney function.

## Supplementary table 4. 1-year and 5-year cumulative incidence for death and major cardiovascular events (MACE) per 100 individuals with 95% confidence intervals (CI) by indication of coronary angiography

|  |  |  |
| --- | --- | --- |
|  |  |  |
|  | **1-year** | **5-year** |
|  |  |  |
|  |  |  |
| **All-cause mortality** |  |  |
| General population | 0.4 (0.3-0.5) | 2.8 (2.5-3.1) |
| SA | 0.7 (0.5-1.0) | 5.1 (4.2-6.2) |
| UA | 1.4 (0.8-2.2) | 7.7 (5.7-10.3) |
| NSTEMI | 4.4 (3.6-5.4) | 12.9 (11.0-15.1) |
| STEMI | 6.4 (5.2-7.9) | 14.8 (12.3-17.7) |
| **MACE** |  |  |
| General population | 0.4 (0.3-0.5) | 2.9 (2.6-3.2) |
| SA | 0.7 (0.5-1.0) | 5.2 (4.3-6.3) |
| UA | 1.4 (0.8-2.3) | 8.2 (6.1-11.0) |
| NSTEMI | 4.8 (3.9-5.8) | 13.4 (11.5-15.7) |
| STEMI | 6.9 (5.6-8.5) | 15.1 (12.6-18.1) |

## Supplementary figure 1. Survival function for all-cause mortality in patients referred to coronary angiography with obstructive CAD compared to an asymptomatic reference population.


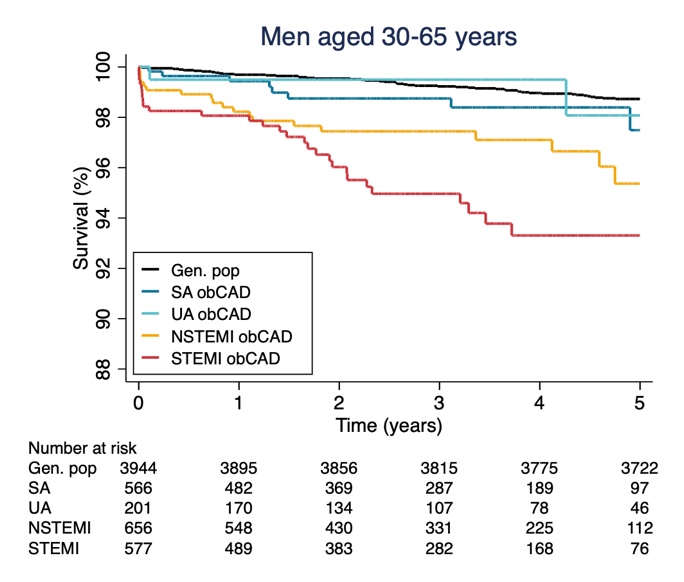

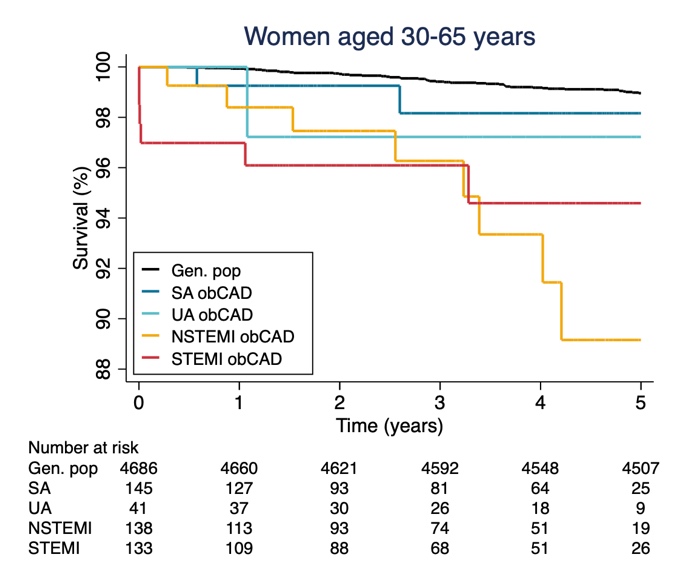


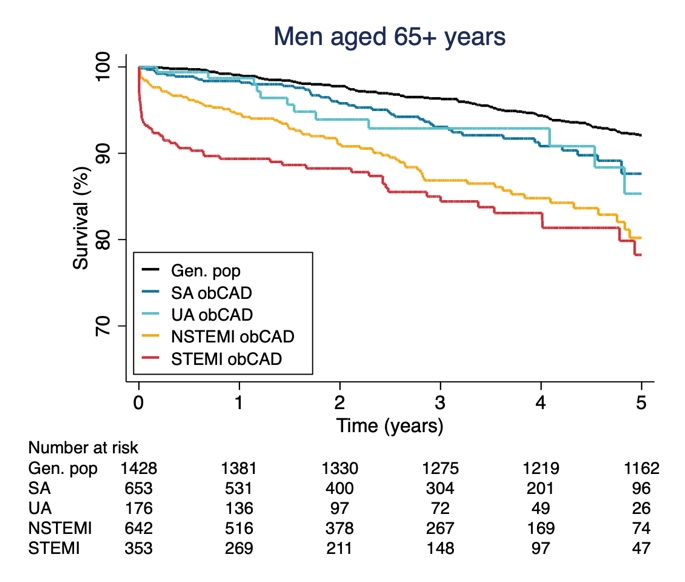

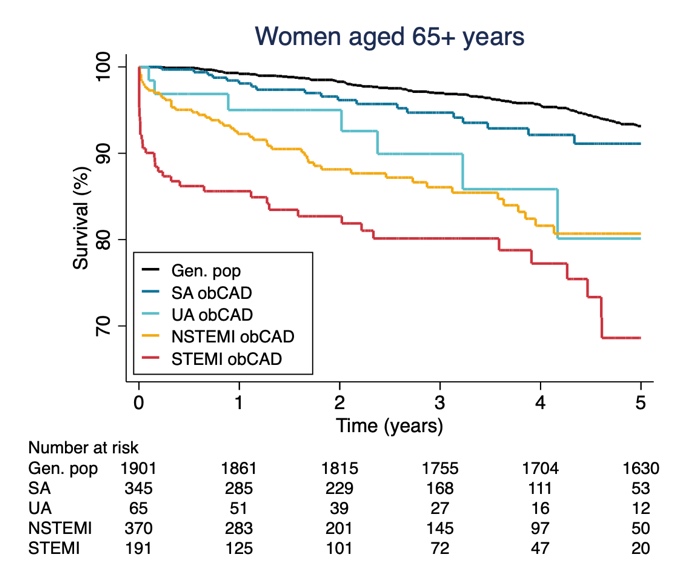


CAD indicates coronary artery disease; obCAD, obstructive CAD; NSTEMI, non-ST segment elevation myocardial infarction; SA, stable angina; STEMI, ST-segment elevation myocardial infarction; UA, unstable angina.

## Supplementary figure 2. Survival function for major adverse cardiovascular events in patients referred to coronary angiography compared to an asymptomatic reference population.


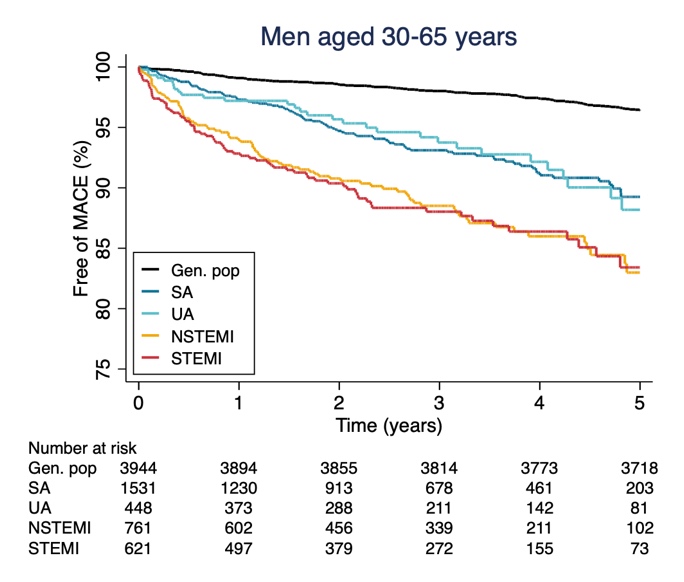

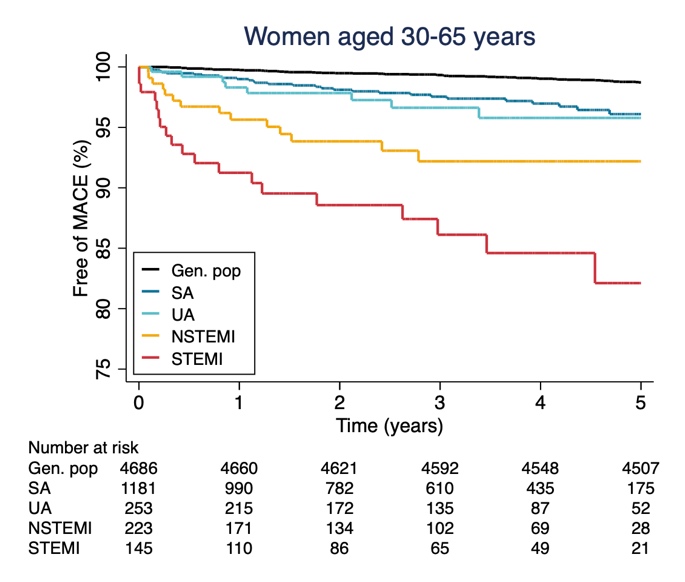


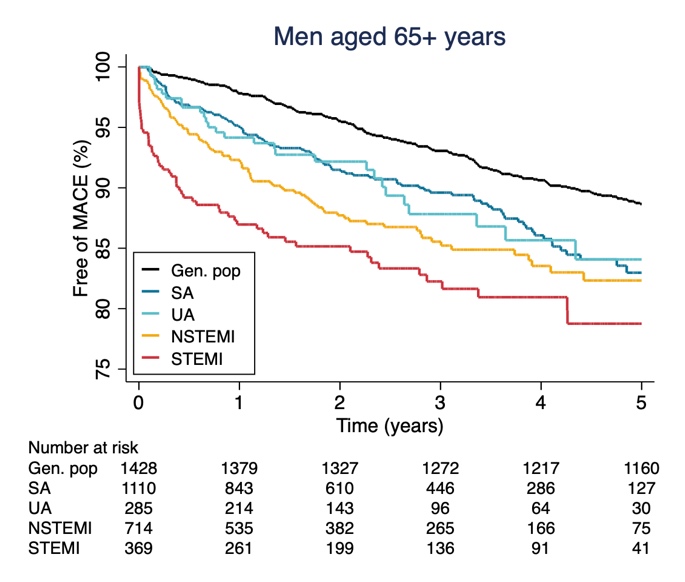

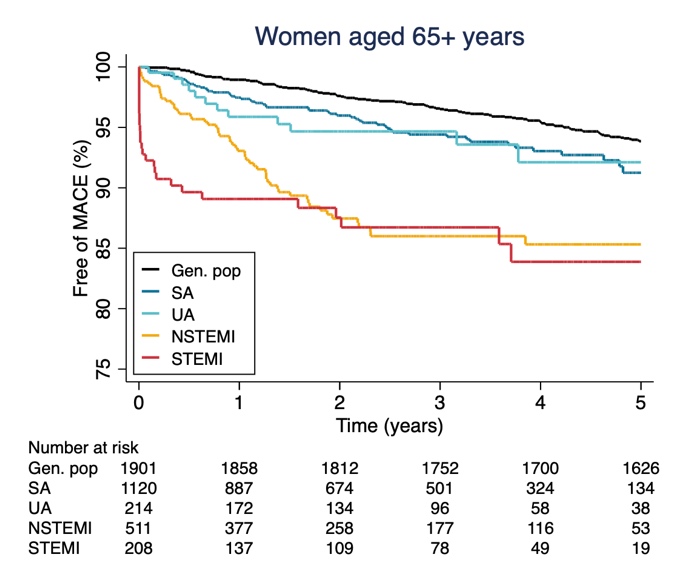


NSTEMI indicates non-ST segment elevation myocardial infarction; SA, stable angina; STEMI, ST-segment elevation myocardial infarction; UA, unstable angina.

## Supplementary figure 3. Survival function for major adverse cardiovascular events for patients referred to coronary angiography by extent of coronary artery disease


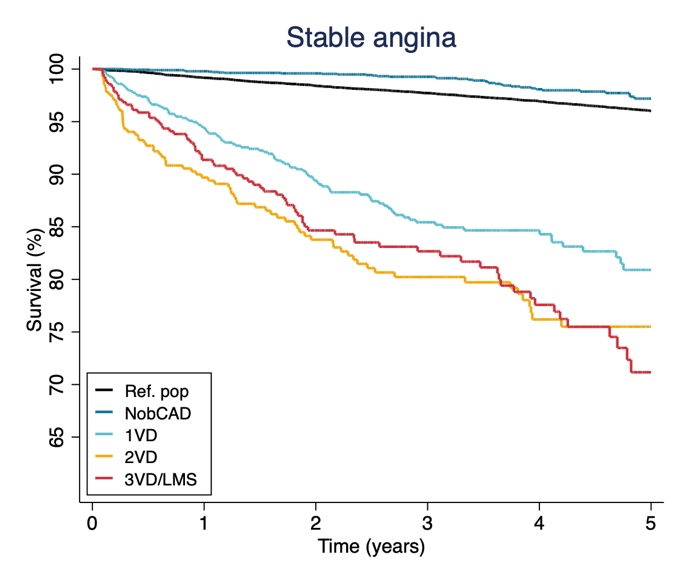

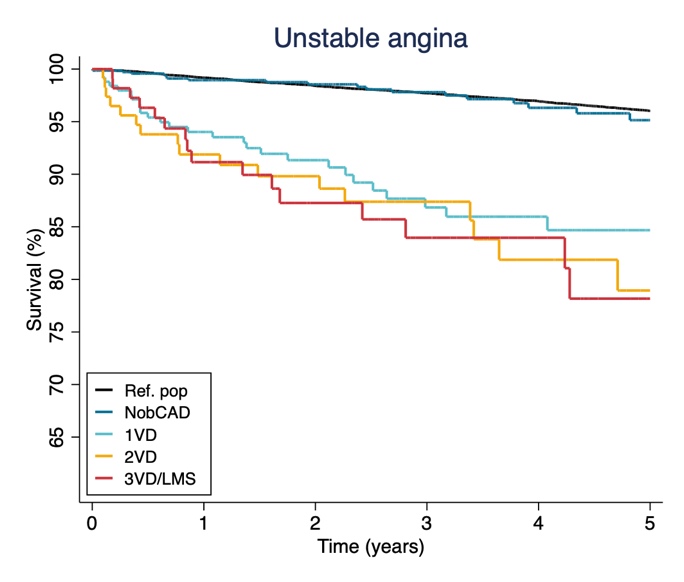


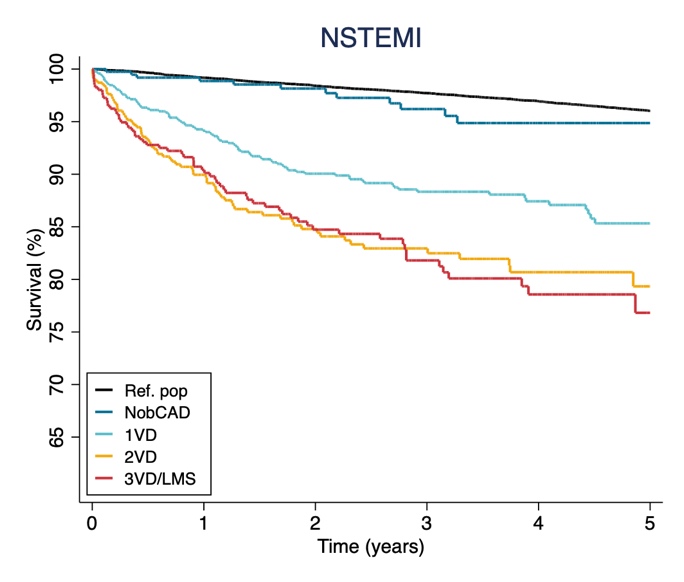

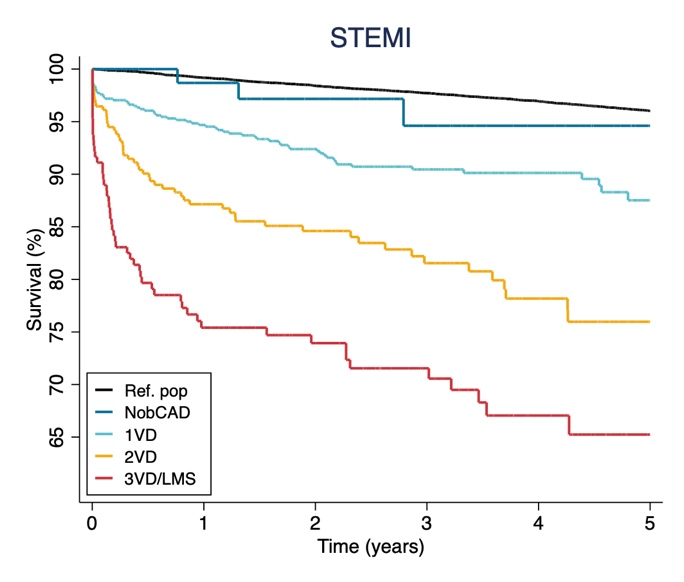


CAD indicates coronary artery disease; non-obCAD, non-obstructive CAD; NSTEMI, non-ST segment elevation myocardial infarction; STEMI, ST-segment elevation myocardial infarction; 1VD, one-vessel disease; 2VD, two-vessel disease; 3VD/LMS, three-vessel disease and/or left main stem disease.
